# MAP4 acts as an oncogene and prognostic marker and affects radioresistance by mediating epithelial–mesenchymal transition in lung adenocarcinoma

**DOI:** 10.1007/s00432-024-05614-8

**Published:** 2024-02-10

**Authors:** Xiaochun Xia, Yangyang Ge, Fanghong Ge, Pei Gu, Yuanyuan Liu, Peng Li, Pengqin Xu

**Affiliations:** 1 Department of Radiation Oncology, Nantong Tumor Hospital, Affiliated Tumor Hospital of Nantong University, Nantong, China; 2 Department of Radiation Oncology, Huaian Hospital of Huaian City, Huaian Cancer Hospital, Huaian, China

**Keywords:** Lung adenocarcinoma, MAP4, Prognosis, Nomogram, Radioresistance, Epithelial–mesenchymal transition

## Abstract

**Purpose:**

To explore the effect of microtubule-associated protein 4 (MAP4) on lung adenocarcinoma cells in vitro and evaluate its prognostic value. Radioresistance, indicated by reduced efficiency of radiotherapy, is a key factor in treatment failure in lung adenocarcinoma (LADC). This study aims to explore the primary mechanism underlying the relationship between MAP4 and radiation resistance in lung adenocarcinoma.

**Methods:**

We analysed the expression of MAP4 in lung adenocarcinoma by real-time quantitative polymerase chain reaction (RT‒qPCR), immunohistochemistry (IHC) and bioinformatics online databases, evaluated the prognostic value of MAP4 in lung adenocarcinoma and studied its relationship with clinicopathological parameters. Cox regression analysis and least absolute shrinkage and selection operator (LASSO) regression analysis identified independent prognostic factors associated with lung adenocarcinoma that were used to construct a nomogram, internal validation was performed. We then evaluated the accuracy and clinical validity of the model using a receiver operating characteristic (ROC) curve, time-dependent C-index analysis, a calibration curve, and decision curve analysis (DCA). Scratch assays and transwell assays were used to explore the effect of MAP4 on the migration and invasion of lung adenocarcinoma cells. Bioinformatics analysis, RT‒qPCR, Cell Counting Kit-8 (CCK-8) assays and Western blot experiments were used to study the relationship between MAP4, epithelial–mesenchymal transition (EMT) and radiation resistance in lung adenocarcinoma.

**Results:**

MAP4 expression in lung adenocarcinoma tissues was significantly higher than that in adjacent normal lung tissues. High expression of MAP4 is associated with poorer overall survival (OS) in patients with lung adenocarcinoma. Univariate Cox regression analysis showed that pT stage, pN stage, TNM stage and MAP4 expression level were significantly associated with poorer OS in LADC patients. Multivariate Cox regression analysis and LASSO regression analysis showed that only the pT stage and MAP4 expression level were associated with LADC prognosis. The nomogram constructed based on the pT stage and MAP4 expression showed good predictive accuracy. ROC curves, corrected C-index values, calibration curves, and DCA results showed that the nomogram performed well in both the training and validation cohorts and had strong clinical applicability. The results of in vitro experiments showed that the downregulation of MAP4 significantly affected the migration and invasion of lung adenocarcinoma cells. MAP4 was strongly correlated with EMT-related markers. Further studies suggested that the downregulation of MAP4 can affect the viability of lung adenocarcinoma cells after irradiation and participate in the radiation resistance of lung adenocarcinoma cells by affecting EMT.

**Conclusion:**

MAP4 is highly expressed in lung adenocarcinoma; it may affect prognosis by promoting the migration and invasion of cancer cells. We developed a nomogram including clinical factors and MAP4 expression that can be used for prognosis prediction in patients with lung adenocarcinoma. MAP4 participates in radiation resistance in lung adenocarcinoma by regulating the radiation-induced EMT process. MAP4 may serve as a biomarker for lung adenocarcinoma prognosis evaluation and as a new target for improving radiosensitivity.

## Introduction

Lung cancer is one of the most common cancers and accounts for the highest number of cancer-related deaths worldwide (Césaire et al. 2022). Lung adenocarcinoma, the most common subtype of lung cancer, is highly aggressive and generally has a poor prognosis (Chen et al. 2018), with a low 5-year survival rate (Chen et al. 2014; Cucchiarelli et al. 2008). Radiation therapy is essential for patients with thoracic malignancies (Bacco et al. 2011). Radiotherapy has obvious benefits for patients who are not good candidates for surgery and is widely used in the radical and palliative treatment of LADC patients (Forges et al. 2012). Approximately 70% of lung cancer patients require radiotherapy to improve local control and survival. However, lung adenocarcinoma is not highly sensitive to radiotherapy, and tumour cells gradually become resistant, often causing local recurrence and metastasis, worsening the prognosis (Dmitriev et al. 2015; Etienne-Manneville 2013). Radiosensitivity is not only determined by tumour histology but also affected by gene pathways (Ettinger et al. 2021). Although radiotherapy is used to improve local control, its overall efficacy is unsatisfactory. Radioresistance is a major factor that reduces the efficacy of radiotherapy and leads to treatment failure. Radioresistance limits the efficacy of radiotherapy. Since the sensitivity of tumour cells is a key factor in the efficacy of radiotherapy, it is necessary to identify key molecules associated with radiosensitivity in lung adenocarcinoma. New biomarkers are needed to predict patient prognosis after radiation therapy and to predict the sensitivity to radiotherapy before treatment.

Microtubule-associated proteins (MAPs) interact with microtubule tubulin dimers. The association of MAPs with microtubules is maintained through the phosphorylation of MAPs (Fu et al. 2019). MAPs occupy key nodes in the cytoskeleton regulatory network. They regulate the organization and function of the microtubule cytoskeleton in interphase and mitosis. Changes in the sequence or expression levels of these proteins may lead to defects in the cytoskeleton that can lead to the initiation or progression of cancer. Indeed, many MAPs have been described as oncogenes or tumour suppressor genes, whose deregulation has a major impact on cancer aggressiveness and clinical outcome in patients. Microtubule dynamics play an important role in several cellular processes, including cell division, cell motility, cell shape maintenance, cell signalling, and intracellular trafficking (Gomez-Casal et al. 2013). Perturbations in microtubule dynamics are closely related to cancer cell behaviour (Győrffy et al. 2014). Microtubules reorganize to promote cancer-related activities, such as mitosis and migration, to promote tumour growth and metastasis, respectively (Győrffy et al. 2014; Hashi et al. 2014). MAP4 is the major MAP protein expressed in nonneuronal mammalian cells and is able to regulate microtubule assembly and stability in vitro and in vivo (Hu et al. 2014). MAP4 has been reported to play key roles in microtubule stabilization and assembly (Hu et al. 2023). Phosphorylation of MAP4 impairs its function and induces its dissociation from microtubules, leading to microtubule depolymerization. Changes in MAP4 phosphorylation levels can affect cell cycle progression (Jiang et al. 2020) and apoptosis (Jiang et al. 2016). To date, little research has been done on the association between MAP4 and human cancer. Studies have shown that stabilization of microtubules by inhibiting the phosphorylation of MAP4 enhances the sensitivity of ovarian cancer cells to paclitaxel. This could be used as a major strategy to improve ovarian cancer treatment efficacy (Kang et al. 2013; Kim et al. 2014). MAP4 has recently been shown to promote cell invasion in solid tumour progression (Kim et al. 2015; Kurrey et al. 2009; Kwon et al. 2017; Lamouille et al. 2014). In terms of prostate cancer, MAP4 has also been considered a potential biomarker for cancer detection and differentiation of prostate tumours with different malignancies and aggressiveness (Li et al. 2016).

Cancer cells can repair radiation-induced damage in a number of ways. This process can cause cells to become radioresistant, eventually leading to tumour recurrence and compromising the efficacy of radiation therapy. Recent studies have shown that EMT plays a crucial role in the development of cancer radioresistance. Radiation can induce EMT, leading to acquired radiation resistance (Li et al. 2020). Epithelial-mesenchymal transition (EMT) is the process by which epithelial cells differentiate into active mesenchymal cells and can contribute to the malignant behaviour of tumour cells following radiation. The main features of EMT include the transition of cell morphology from an epithelial phenotype to a mesenchymal phenotype; in this process, epithelial cells lose cell polarity and adhesion ability but acquire cell motility, that is, mesenchymal stem cell characteristics. EMT is characterized by the downregulation of E-cadherin (marker of epithelial cells) and the upregulation of N-cadherin and vimentin (markers of mesenchymal cells). EMT is also a hallmark of tumorigenesis and progression. It is well known that EMT endows tumour cells with a stronger metastatic ability (Li et al. 2017; Li et al. 2015). Long-term exposure to radiation or chemotherapeutic drugs, including cisplatin and different taxanes, can lead to spindle morphogenesis and increased expression of EMT-related proteins in NSCLC cells (Li et al. 2021). Understanding the mechanisms underlying EMT radiation resistance may aid in the development of more effective therapeutic drugs and treatments.

However, the therapeutic value and possible mechanisms of MAP4 in LADC remain to be investigated. There is no experimental evidence to prove the relationship between MAP4 and tumour radiosensitivity. In this study, we hypothesized that MAP4 is an independent prognostic factor and radiosensitizing target in patients with lung adenocarcinoma. The lung adenocarcinoma cell lines A549 and H1299 were used to study the effect of inhibiting MAP4 gene expression on radiosensitivity and to determine its preliminary mechanism related to EMT regulation. Ultimately, this study provides important support for MAP4 as a target for increasing sensitivity to radiotherapy.

## Materials and methods

### Patients, tissue samples and dataset

In total, 270 LADC patients undergoing curative resection at Nantong Tumour Hospital from January 2010 to December 2020 were included in this study. No patient had received any antitumour therapy before surgery. Twenty-seven pairs of lung adenocarcinoma tissues and tumour-adjacent lung tissues were obtained in this study. The exclusion criteria were as follows: (1) pathological stage I–III lung adenocarcinoma; (2) patients undergoing surgery for LADC; (3) complete clinical and pathological information and auxiliary examination results available; and (4) complete follow-up information. The exclusion criteria were as follows: (1) incomplete clinical data; (2) local or systemic antitumour therapy before surgery; (3) distant metastasis; and (4) other malignancy histories. The included patients were randomly divided into training (*n *= 180) and validation cohorts (*n* = 90) at a ratio of 2:1. Three datasets (243_g_at, 200836_s_at, 212567_g_at) of LADC subjects were retrieved from the K‒M plotter web-based survival analysis tool (https://kmplot.com/analysis/) (Lu and Kang 2019). A preliminary analysis of the correlation between MAP4 protein expression and EMT markers was performed on 515 cases of lung adenocarcinoma using TIMER (https://cistrome.shinyapps.io/timer/) (Luo et al. 2021; Luo et al. 2019). Since the three LADC datasets were from an open database, informed consent was not used. This retrospective analysis of anonymous data was approved by the institutional ethics review boards of Nantong Tumor Hospital, and informed consent was waived by the ethics review boards.

### Immunohistochemistry

Conventional paraffin-embedded samples were precooled, and LADC tissue samples and adjacent tissues were cut into 4 μm thick slices and pasted onto slides. The samples were treated by xylene dewaxing 3 times and ethanol gradient dehydration. The sections were immersed in EDTA (pH 8) and autoclaved at 121 °C for 5 min to restore antigenicity. After cooling to room temperature, the sample was sealed in 3% hydrogen peroxide methanol solution for 10 min to eliminate endogenous peroxidase activity. MAP4 antibodies (Proteintech, Rosemont, IL, US) diluted at 1:4000 were incubated overnight at 4 °C, followed by further washing with buffers to remove unbound antibodies. At room temperature, the slices were incubated with Envision secondary antibody (DAKO, Santa Clara, CA, USA) for 30 min. The sample was cleaned with phosphate buffer solution (PBS) 3 times. Chromogen (DAB) (GeneTex, Irvine, CA, US) was used for colour development. Haematoxylin (Beyotime Biotechnology, Nantong, Jiangsu, China) was used for tissue restaining. Finally, the sections were dehydrated in alcohol and xylene and sealed. The results were observed under an optical microscope, and the images were analysed. Immunohistochemical staining evaluation was performed as described previously (Kwon et al. 2017).

### Cell culture and irradiation

A549 and H1299 cell lines were purchased from the Shanghai Cell Bank (Shanghai, China). A549 cells were cultured in DMEM, and H1299 cells were cultured in RPMI-1640 medium supplemented with 10% foetal bovine serum (FBS) (HyClone, Waltham, MA, US) containing double resistance (1%). All cell lines were incubated in a humidified atmosphere with 5% CO_2_ at 37 °C. Cells were exposed to different dosages of ionizing radiation using an X-ray linear accelerator (Elekta Synergy, Stockholm, Sweden) at a fixed dose rate of 2 Gy/min.

### siRNAs and real-time qPCR

MAP4-specific targeted siRNA was obtained from Ruibo Biotechnology (Ribobio, Guangzhou, Guangdong, China). The siMAP4 sequence is 5'-CCAACGTGATCTTGACCAA-3'. When the cells reached 30% confluency, siRNA was transfected into the cells using Lipofectamine 3000 (Invitrogen, Carlsbad, CA, USA). Total RNA was extracted using TRIzol reagent (Invitrogen, Carlsbad, CA, USA). The detailed experimental procedure has been described previously (Kwon et al. 2017). β-Actin was used as the internal reference. All samples were examined in triplicate. The expression of RNA was analysed and quantified by the 2 − ΔΔCt method.

### Wound healing (scratch) assay

Lung adenocarcinoma cell lines were seeded into 6-well plates. When the cells had grown to confluence, five scrapes were scratched vertically with a sterile 1000 µL pipettor tip. Pictures were taken after washing three times with PBS. Cell migration at 0 and 48 h was photographed under an inverted phase contrast microscope (Leica Corporation, Wetzlar, Germany), and the width of the wound area was measured. Wound healing or cell migration rates were judged by the cell-free area at 0 and 48 h.

### Cell invasion assay

A total of 1 × 10^5^ cells were transferred to an apical chamber equipped with a serum-free medium. Transwell inserts were precoated with 40 μl of Matrigel (BD Biosciences, San Jose, CA, US). Serum-containing medium (10% FBS) was used as a chemoattractant in the lower chamber. Cells were also incubated for 24 h at 37 °C. Subsequently, we removed noninvasive cells at the top of the membrane by washing and then fixed the migrating cells with 3.7% paraformaldehyde and stained them with 2% crystal violet. The total number of invaded cells was counted under a light microscope. Counts were performed in three random fields at 200 × magnification.

### Cell counting Kit-8 (CCK-8) assay

A total of 5*10^3^ cells/well were seeded in 96-well plates, and the cultured A549 and H1299 cells were subsequently treated with IR and IR plus MAP4 siRNA. Cell viability was analysed by the CCK8 assay (Beyotime Biotechnology, Nantong, Jiangsu, China). Cells were incubated with 10 µL of CCK-8 solution at 37 °C for 2 h. The absorbance of the mixture was measured at 450 nm with BioTek ELX808.

### Western blotting

Total proteins in the cells were extracted with RIPA lysis buffer containing protease inhibitors and quantified with the BCA Protein Assay kit (Beyotime Biotechnology, Nantong, Jiangsu, China). Sample proteins were separated by 10% SDS‒PAGE and transferred to PVDF membranes (Millipore, Bedford, MA, US). After blocking with 5% skim milk in TBS-Tween-20 for 1 h at room temperature, the membranes were incubated with primary antibodies against GAPDH (Beyotime Biotechnology, Nantong, Jiangsu, China), E-cadherin, N-cadherin, Vimentin, Slug (Abcam Biotechnology, Cambridge, UK) and MAP4 (Proteintech, Rosemont, IL, US). They were then incubated with primary antibodies overnight at 4 ℃. After washing three times with TBST, the membranes were incubated with anti-mouse or anti-rabbit horseradish peroxidase-labelled secondary antibodies for 2 h at room temperature. Proteins were visualized using an Enhanced Chemiluminescence (ECL) Western blot Analysis System (Bio-Rad). All data were normalized to GAPDH levels.

### Construction and validation of nomograms

A total of 270 patients were allocated to training and validation cohorts in a 2:1 ratio. The training cohort was designated for the construction of the nomogram, while the validation cohort was used for internal validation of the model. Factors predicting the prognosis of lung adenocarcinoma include age at diagnosis, sex, smoking history, degree of differentiation, T stage, N stage, TNM stage, and MAP4 expression. Univariate and multivariate Cox proportional hazards regression analyses were used to assess prognostic factors for LADC. LASSO regression was performed to determine independent prognostic indicators for LADC. Only variables with nonzero coefficients and optimal regularization parameter values were used to construct 1-, 3-, and 5-year OS nomograms. Tenfold cross-validation was used to optimize regularization parameters.

### Assessment of the performance of the different models

To assess the accuracy of the nomogram, the time-dependent area under the ROC curve (AUC) and the time-dependent C-index were used to verify the predictive performance of the nomogram in the training and validation cohorts. The C-index is used to measure the accuracy of model predictions. A C-index value higher than 0.7 indicates that the predictive model has excellent discriminative ability. The adjusted C-index was calculated using bootstrap resampling validation (using 1000 bootstrap resamples). An AUC greater than 0.7 indicates that the model has excellent discriminative ability. In addition, we used calibration curves to assess the agreement between actual outcomes and predicted probabilities. A curve close to the 45-degree diagonal indicates that the predicted probabilities agree with the actual observed probabilities. DCA was used to assess the net clinical benefit of the nomogram and the clinical utility of the nomogram.

### Statistical analysis

R statistical software (version 4.2.2; R Foundation for Statistical Computing, Vienna, Austria; https://www.r-project.org/) and GraphPad Prism 8 (GraphPad Software, La Jolla, CA, US) were used for statistical analysis. The R packages used in this study included “survival” and “survminer” (for survival curves), “glmnet” (for LASSO regression), “forestplot” (for forest plots), “rms” (for nomograms and calibration curve), “pROC” and “timeROC” (for ROC curves) and “ggDCA” (for DCA). Student's t test was used to compare quantitative data. Paired samples were compared using a paired sample t test. Count data were analysed by the chi-square test and expressed in *n* (%). Survival curves were drawn using the Kaplan‒Meier method. The log-rank test was used to compare the OS curves of different groups. Univariate and multivariate analyses of survival were performed using Cox proportional hazards regression models. All statistical tests were two-sided, and *P* < 0.05 was considered statistically significant.

## Results

### Patient characteristics

A total of 270 patients were included, including 147 (54.44%) males and 123 (45.56%) females, and 141 (52.22%) of them were over 60 years old. The median follow-up was 49 months (range 1–121 months). There were 166 patients (61.48%) with a smoking history. In addition, 69 (25.56%) were poorly differentiated, and another 201 (74.44%) were moderately or well differentiated. According to the TNM staging system, 169 (62.59%) and 101 (37.41%) LADC patients were diagnosed with stage II-III and stage I disease, respectively. Among them, 213 (78.89%) were in T1-2 stage, and 57 (21.11%) were in T3-4 stage. A total of 135 (50.00%) patients had lymph node metastasis. MAP4 immunohistochemical staining scores > 4 were considered indicative of high expression levels. There were 151 patients (55.92%) with high expression of MAP4 and 119 patients (44.08%) with low expression of MAP4. Table 1 summarizes the baseline demographic and clinical characteristics of all patients. The distribution of these baseline variables in the training cohort (*n* = 180) and the validation cohort (*n* = 90) was well balanced (*P* > 0.05).

**Table 1:** The clinical characteristics of patients with lung adenocarcinoma in training and validation cohorts

Variable	Overall (*n* = 270, %)	Training cohort (*n* = 180, %)	validation cohort (*n* = 90, %)	*χ*^2^	*P* value
Gender				0.07	0.80
Male	147 (54.44)	97 (53.89)	50 (55.56)		
Female	123 (45.56)	83 (46.11)	40 (44.44)		
Age (year)				0.00	1.00
> 60	141 (52.22)	94 (52.22)	47 (52.22)		
≤ 60	129 (47.78)	86 (47.78)	43 (47.78)		
Smoke				0.12	0.72
Yes	166 (61.48)	112 (62.22)	54 (60.00)		
No	104 (38.52)	68 (37.78)	36 (40.00)		
Differentiation				0.09	0.77
Poor	69 (25.56)	47 (26.11)	22 (24.44)		
Well/moderate	201 (74.44)	133 (73.89)	68 (75.56)		
pT stage				2.50	0.11
T3-4	57 (21.11)	33 (18.33)	24 (26.67)		
T1-2	213 (78.89)	147 (81.67)	66 (73.33)		
pN stage				1.67	0.20
N1-3	135 (50.00)	85 (47.22)	50 (55.56)		
N0	135 (50.00)	95 (52.78)	40 (44.44)		
TNM stage				3.16	0.08
I–II	169 (62.59)	106 (58.89)	63 (70.00)		
I	101 (37.41)	74 (41.11)	27 (30.00)		
MAP4 expression				0.01	0.93
High	151 (55.92)	101 (56.11)	50 (55.56)		
Low	119 (44.08)	79 (43.89)	40 (44.44)		

### High expression of MAP4 is associated with a poor prognosis in lung adenocarcinoma patients

**Fig. 1:**
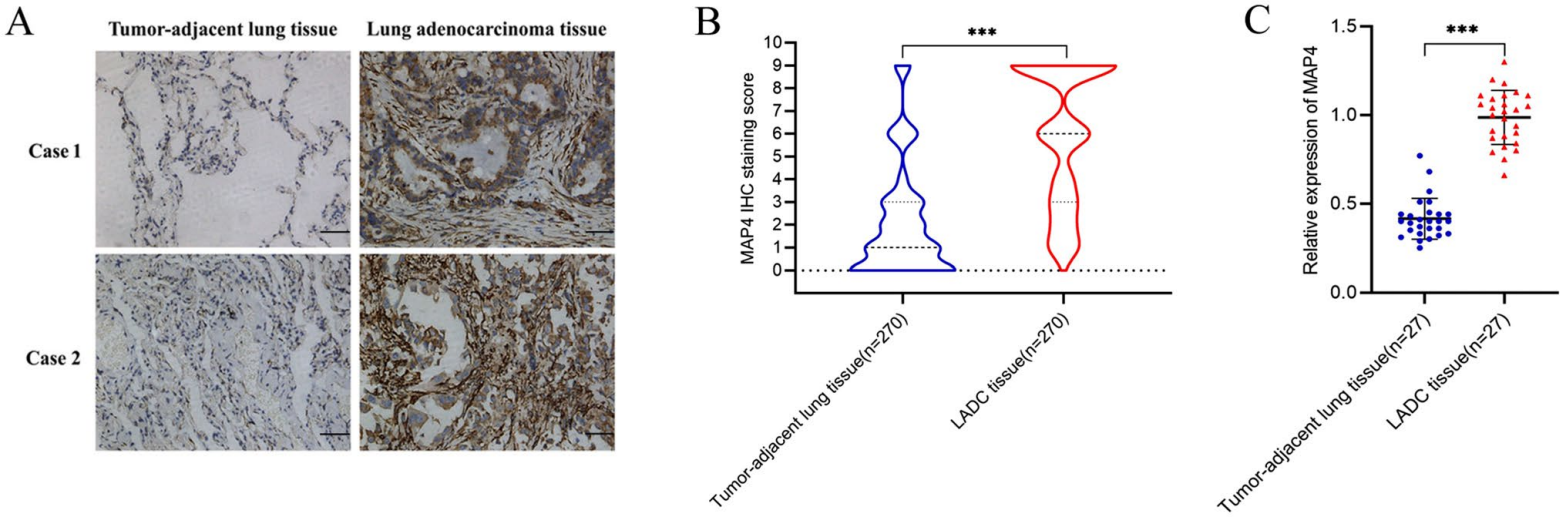
High expression of MAP4 in lung adenocarcinoma tissue samples. **A** Representative immunohistochemical (IHC) staining of MAP4 in tumour-adjacent lung tissue and lung adenocarcinoma tissues (magnification, 200X). **B** Violin chart representing the range of MAP4 IHC staining scores in tumour-adjacent lung tissues and lung adenocarcinoma tissues (*n* = 270). **C** Detection of MAP4 expression in tumour-adjacent lung tissues and lung adenocarcinoma tissues via RT‒qPCR

**Fig. 2:**
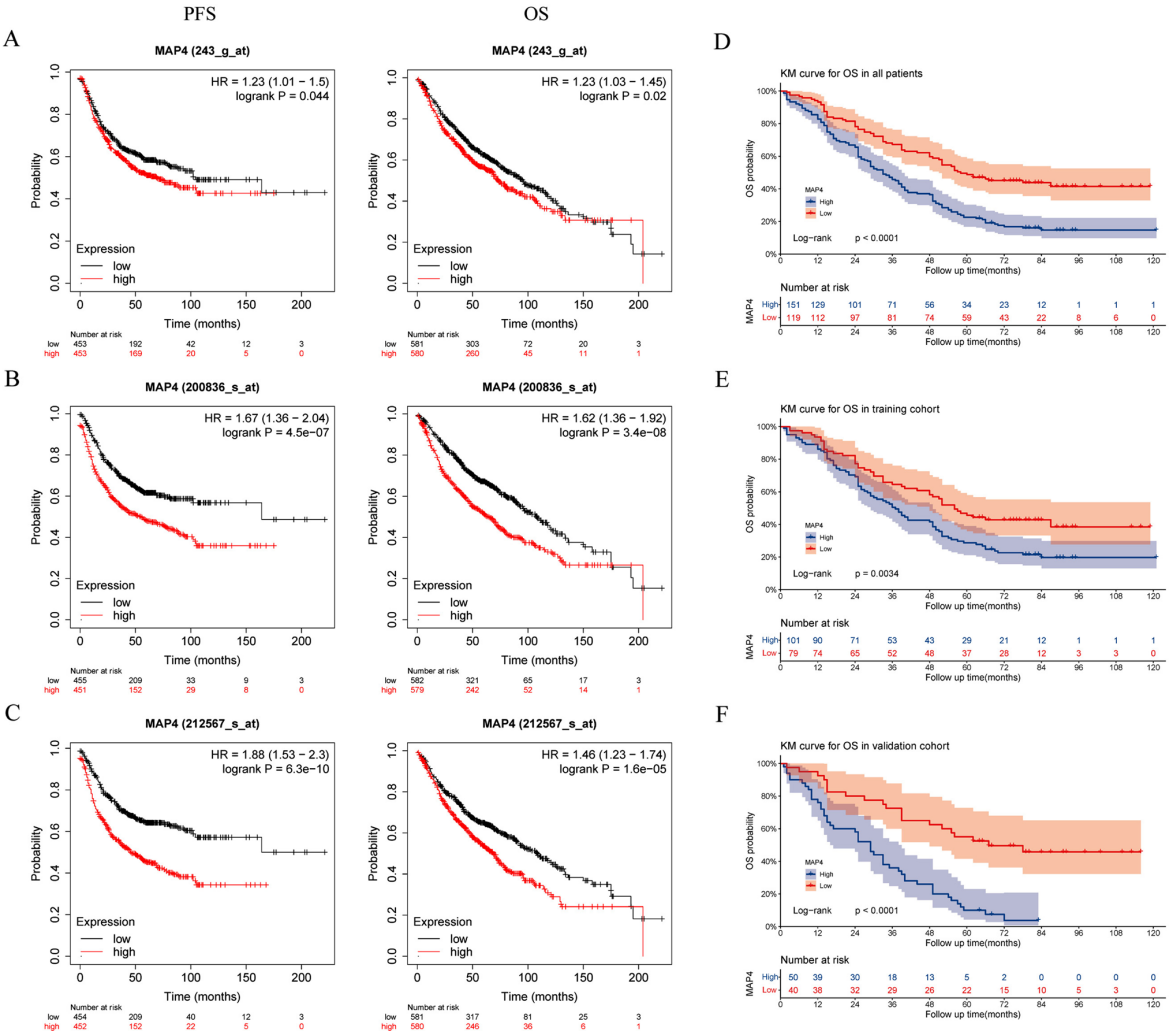
High MAP4 expression correlates with poor prognosis in lung adenocarcinoma patients. PFS and OS curves of MAP4 expression in the 243_g_at (**A**), 200836_s_at (**B**) and 212567_g_at (**C**) datasets from the K‒M plotter web-based survival analysis tool. Kaplan‒Meier overall survival curves were applied for all patients (**D**), the training cohort (**E**) and the validation cohort (**F**) with different MAP4 expression levels, and the log-rank test was used to calculate *p* values

Immunohistochemical analysis was performed on lung adenocarcinoma tissues and adjacent lung tissues to determine the expression of MAP4 in lung adenocarcinoma tissues. The results showed that MAP4 staining was stronger in lung adenocarcinoma tissues than in adjacent noncancerous tissues. There were 151 (55.92%) patients with lung adenocarcinoma who highly expressed MAP4. Figure 1A shows the immunohistochemical expression of MAP4 in lung adenocarcinoma tissue and adjacent normal lung tissue of two typical patients. The results of immunohistochemical scoring suggested that the expression of MAP4 in lung adenocarcinoma tissue was significantly higher than that in adjacent normal lung tissue (Fig. 1B, *P* < 0.001). Likewise, the RT‒qPCR results showed that the expression level of MAP4 in lung adenocarcinoma tissues was significantly higher than that in adjacent normal lung tissues (Fig. 1C, *P* < 0.001). The results of bioinformatics analysis suggested that in the three datasets of 243_g_at (Fig. 2A), 200836_s_at (Fig. 2B) and 212567_g_at (Fig. 2C), the expression level of MAP4 was negatively correlated with the progression-free survival (PFS) and overall survival (OS) times of lung adenocarcinoma patients (Fig. 2D). Kaplan‒Meier survival curves showed that high expression of MAP4 was associated with poor OS in patients with lung adenocarcinoma in the overall population (Fig. 2D, *P*< 0.0001), training cohort (Fig. 2E, *P* = 0.0034) and validation cohort (Fig. 2F,  *P* < 0.0001). All the results indicated that patients with high expression of MAP4 in lung adenocarcinoma had poorer overall survival.

### Determination of independent prognostic factors for LADC

**Fig. 3:**
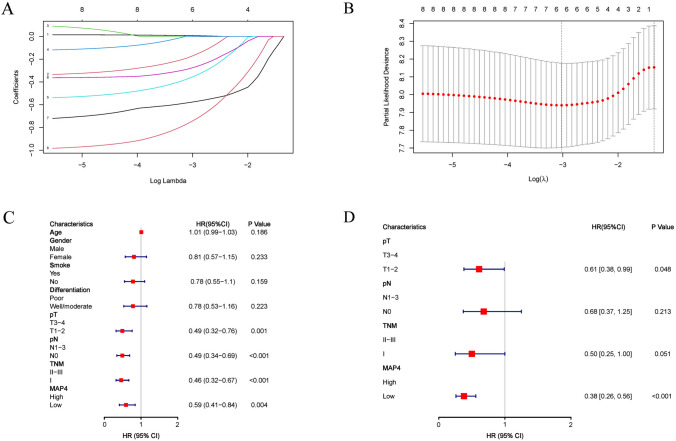
The relationship between MAP4 expression, clinical parameters and OS in LADC. LASSO regression analysis identified the risk factors for OS in LADC (**A**, **B**). Forest plot of univariate (**C**) and multivariate (**D**) Cox regression analyses for OS in lung adenocarcinoma patients in the training cohort

To investigate the prognostic value of differential expression of MAP4 in LADC patients, we performed univariate Cox proportional hazards regression analysis. Univariate analysis showed that pT stage (*P* = 0.001), pN stage (*P* < 0.001), TNM stage (*P* < 0.001) and MAP4 expression level (*P* = 0.004) were significantly associated with poorer OS in LADC (Fig. 3C). Further multivariate regression analysis showed that pT stage (*P* = 0.048) and MAP4 expression level (*P* < 0.001) were independent prognostic factors for the overall survival of patients with lung adenocarcinoma (Fig. 3D). Further screening by LASSO regression analysis showed that, consistent with multivariate Cox regression results, only pT stage and MAP4 expression level were found to be independently associated with LADC prognosis (Fig. 3A, B).

### Establishment and validation of the nomogram

**Fig. 4:**
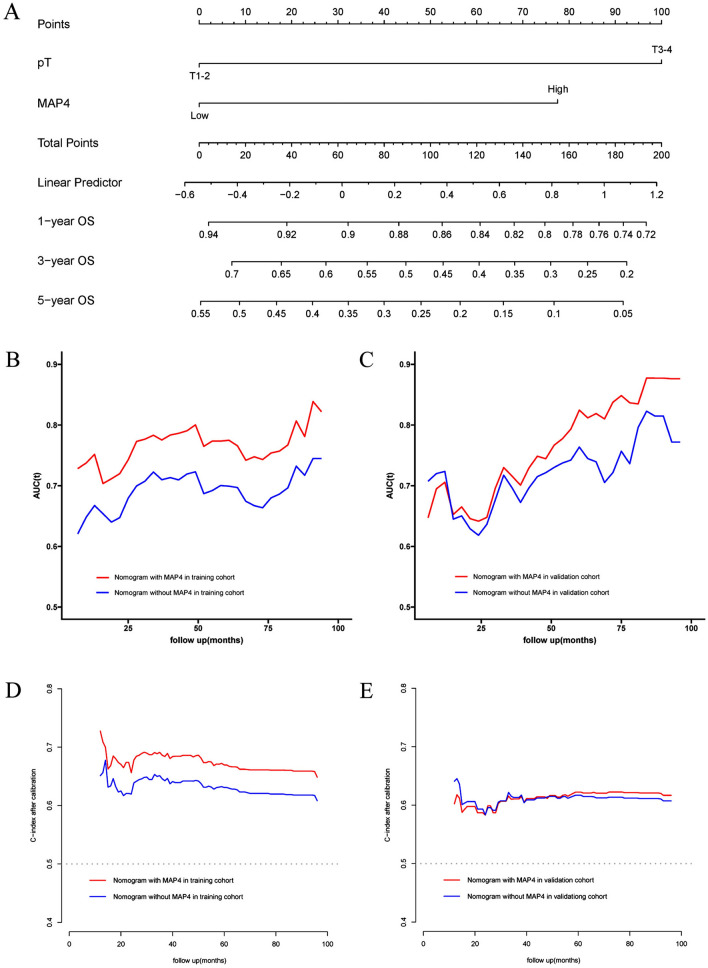
Visualization of OS models based on MAP4 expression combined with clinical characteristics. The nomogram combined with the independent poor prognostic factors, including MAP4 expression and pT stage, to predict the risk of OS at 1, 3, and 5 years (**A**). Time-dependent AUC curves of the nomogram with MAP4 and without MAP4 for the prediction of OS in the training (**B**) and validation cohorts (**C**). Time-dependent C-index curves of the nomogram with MAP4 and without MAP4 for the prediction of OS in the training (**D**) and validation cohorts (**E**)

**Fig. 5:**
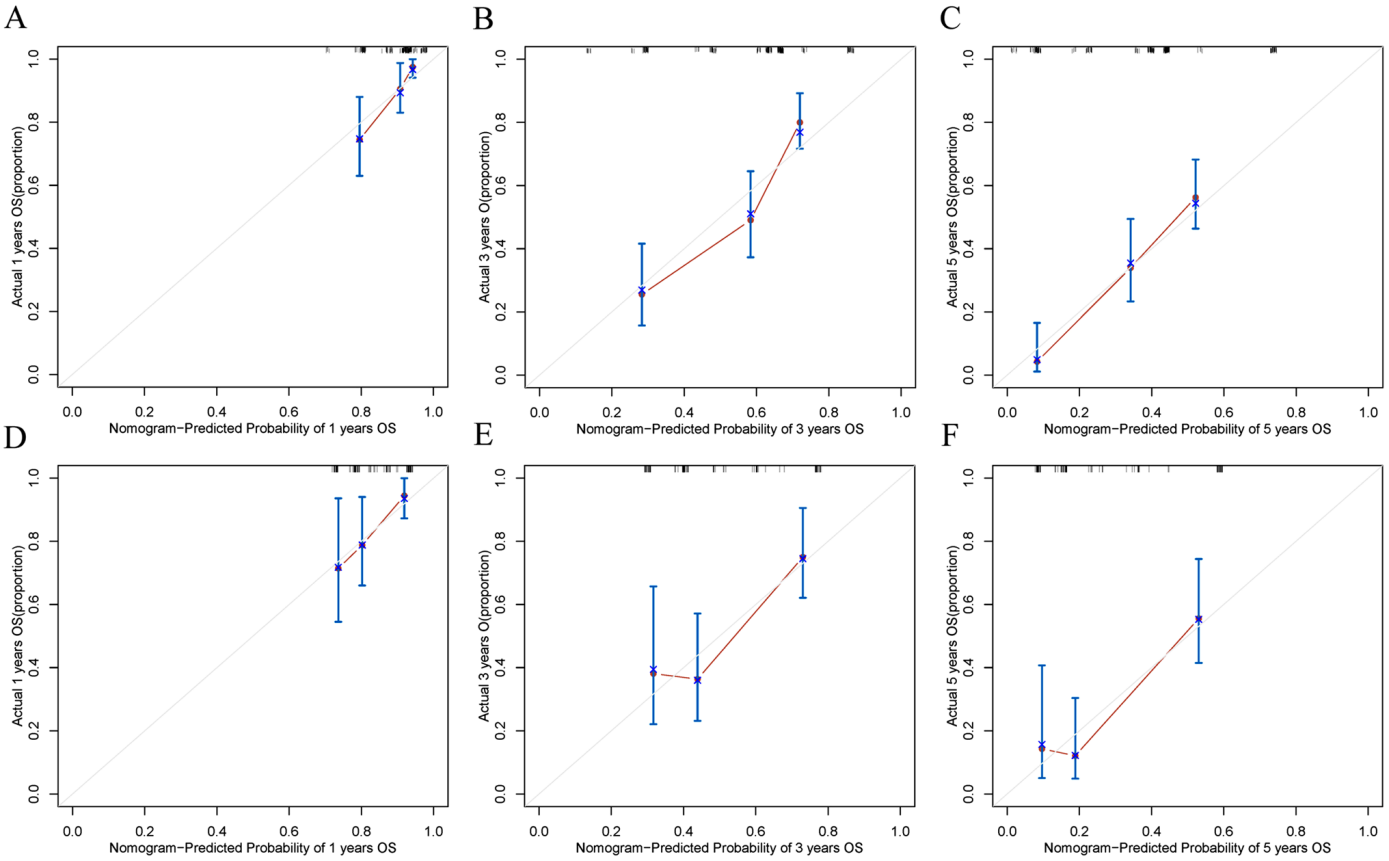
Calibration curve analysis for the nomogram of OS at 1, 3, and 5 years. The calibration curves for the nomogram with the training cohort (**A**: 1-year OS; **B**: 3-year OS; **C**: 5-year OS) and the validation cohort (D: 1-year OS; E: 3-year OS; F: 5-year OS). The 45° diagonal line represents the ideal state, and the solid red line represents the actual predictive value. A closer distance between two curves indicates higher accuracy

**Fig. 6:**
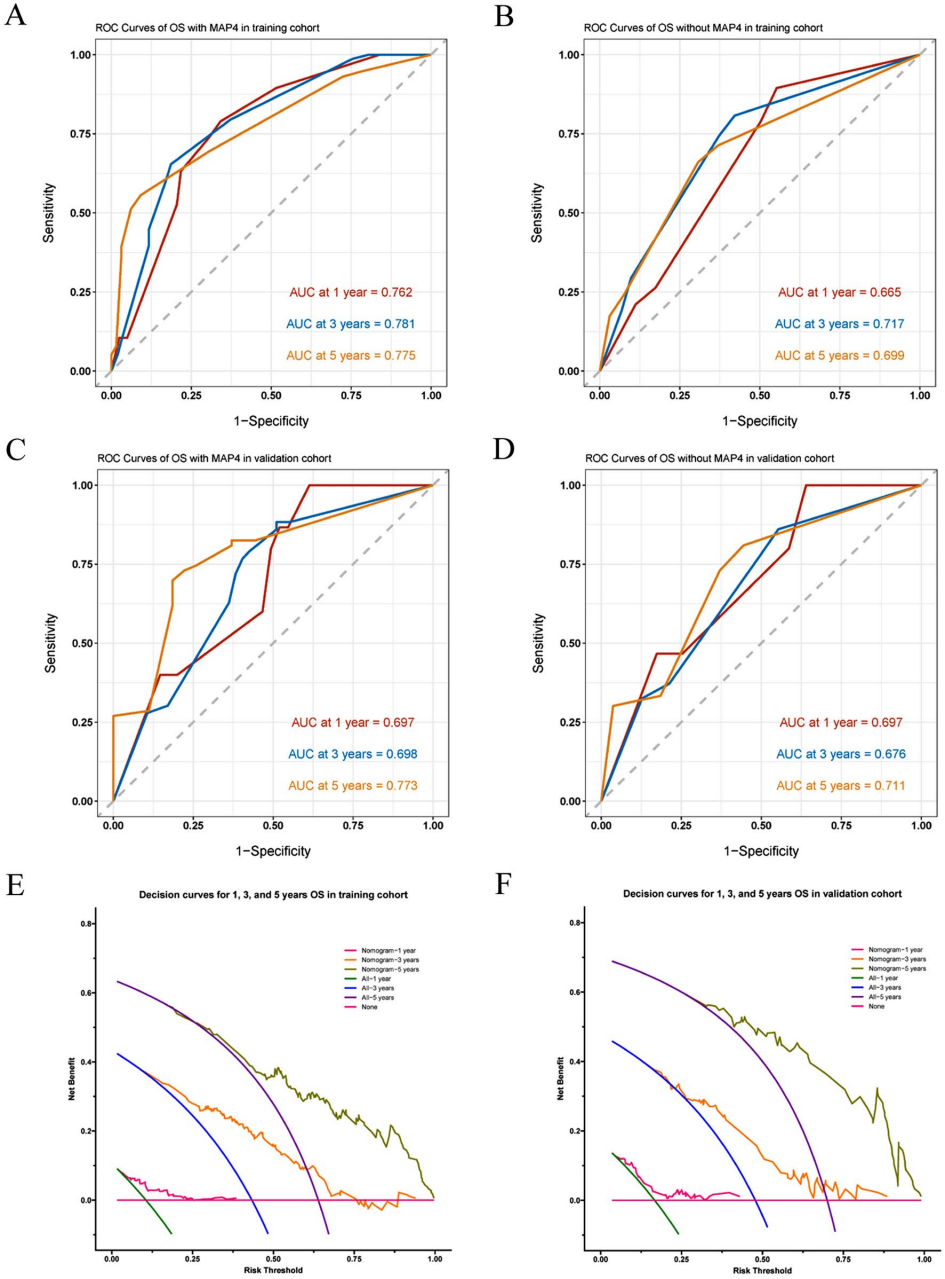
ROC curve and decision curve analysis for the nomogram of OS at 1, 3, and 5 years. ROC curve with MAP4 (**A**, **C**) and without MAP4 (**B**, **D**) for nomogram of OS probability in the training cohort and in the validation cohort, respectively. The addition of MAP4 improved the predictive ability of the nomogram for OS in the study cohort. DCA curves of the nomogram to predict the 1-, 3-, and 5-year OS in the training cohort (**E**) and the validation cohort (**F**). DCA curves showed that the model with MAP4 benefited patients in the prediction of OS at 1, 3, and 5 years

According to the results obtained by LASSO regression analysis, pT stage and MAP4 expression level were incorporated into the nomogram (Fig. 4A). The C-index was used to assess the discriminative ability of the nomogram model. The closer the C-index is to 1, the better the predictive ability of the model for prognosis. According to its predictive ability, the time-dependent AUC curves and the time-dependent calibrated C-index curves were plotted to compare the predictive accuracy of the nomogram. The results suggested that the AUC value (Fig. 4B) and C-index (Fig. 4D) of the nomogram constructed based on MAP4 expression and clinical factors in the training cohort were significantly higher than those of the nomogram constructed without MAsP4. However, the values were lower in the validation cohort than in the training cohort (Fig. 4C, E). Calibration curves were used to assess the agreement of model predictions with actual observations. The closer the curve is to a diagonal line, the closer the predicted probability is to the actual probability, indicating that the nomogram has good reproducibility and reliability. Calibration curves for 1-year, 3-year, and 5-year OS rates, both in the training cohort (Fig. 5A–C) and the validation cohort (Fig. 5D–F), showed that the nomogram predictions were in good agreement with actual observations. Figure 6A–D shows the ability of nomograms with and without MAP4 to predict 1-, 3-, and 5-year OS in the training and validation cohorts. The results suggest that the nomogram constructed in combination with MAP4 has a higher AUC value and predictive ability. Furthermore, we performed DCA and found that our MAP4-based nomogram model achieved a high net benefit in predicting 1-, 3-, and 5-year OS (Fig. 6E–F).

### MAP4 promotes LADC cell migration and invasion

**Fig. 7:**
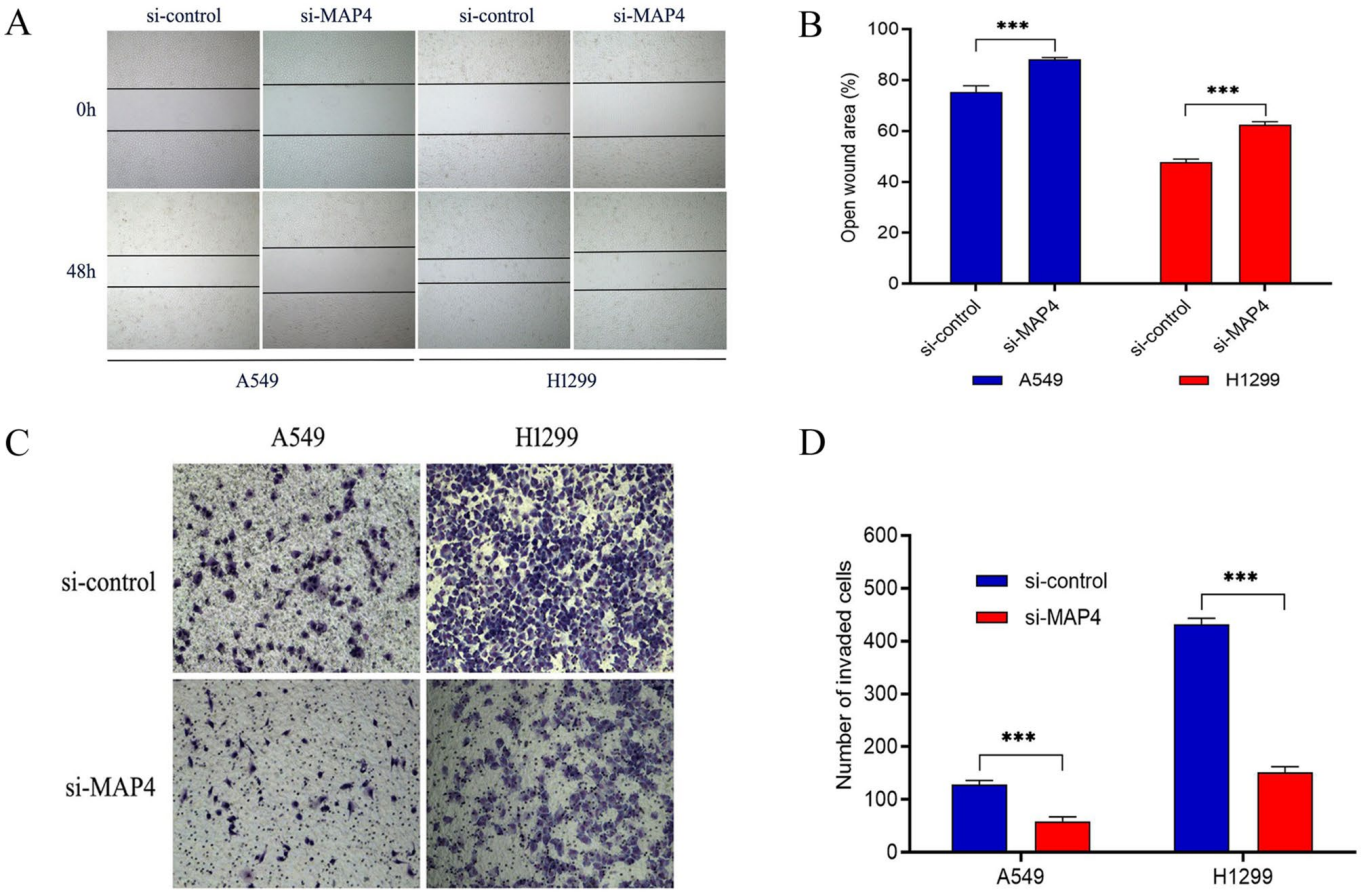
MAP4 promotes the migration and invasion of lung adenocarcinoma cell lines. **A**, **B** The influence of MAP4 expression on lung adenocarcinoma cell migration was determined by wound healing assay in A549 and H1299 cells. The open wound area was normalized to the area at the initial time (0 h). **C**, **D** The effects of MAP4 expression on cell invasion detected by Transwell assay. Data are expressed as the mean ± SD of different groups of cells from three separate experiments. ***P < 0.001

To determine the effect of MAP4 on the migration and invasion of lung adenocarcinoma cells, MAP4 small interfering RNA was used to transiently downregulate MAP4 expression. The results of scratch experiments suggested that knockdown of MAP4 significantly slowed the migration of A549 and H1299 cells compared with control cells (all *P* < 0.001, Fig. 7A, B). In addition, the effects of MAP4 on cell invasion were detected by transwell assay, and the results showed that the invasion activity of A549 and H1299 cells was significantly inhibited after interfering with MA P4 expression (all *P* < 0.001, Fig. 7C, D). Taken together, these results suggest that M AP4 promotes the migration and invasion of lung adenocarcinoma cells.

### MAP4 affects the radiosensitivity of lung adenocarcinoma cells through EMT

**Fig. 8:**
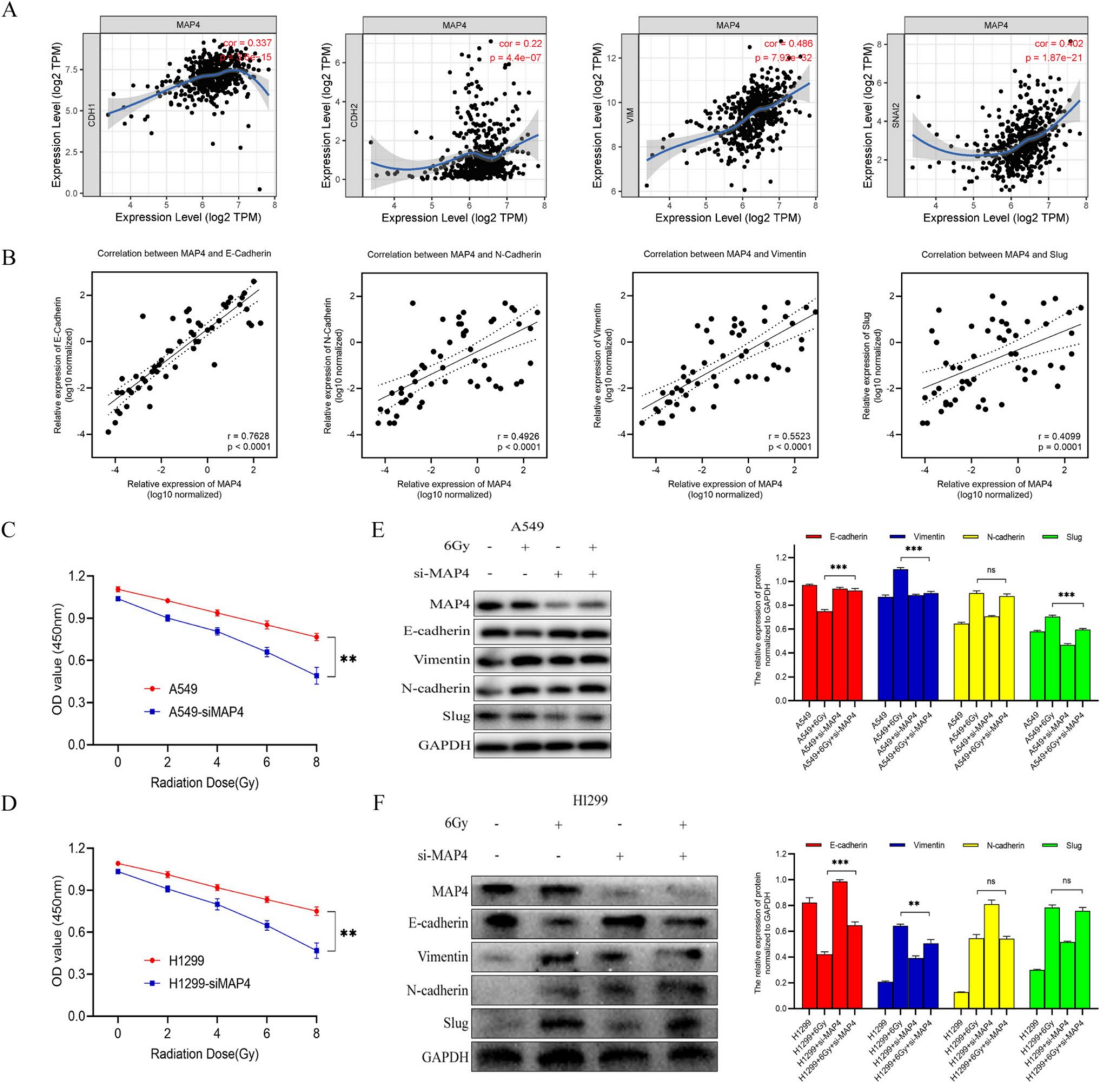
Correlations of the protein levels of MAP4 with those of E-cadherin (CDH1), N-cadherin (CDH2), Vimentin (VIM) and Slug (SNAI2) in 515 cases of lung adenocarcinoma (TCGA) from the TIMER bioinformatics database (**A**). *E*-cadherin, *N*-cadherin, Vimentin, Slug and MAP4 expression was detected in 54 LADC tissues by RT‑qPCR. MAP4 expression was positively correlated with EMT markers (**B**). Cell proliferation evaluated by the CCK8 assay showed that IR plus siMAP4 suppressed cell growth in an IR dose-dependent manner in A549 (**C**) and H1299 (**D**) cells. A549 (**E**) and H1299 (F) cells treated with MAP4 siRNA were exposed to 6 Gy radiation. The expression of EMT-related markers in response to IR treatment was detected by Western blotting. The expression of target proteins was normalized to that of GAPDH and represented as the mean ± SD relative to the control from three separate experiments. **P < 0.01, ***P < 0.001

Due to limited MAP4 expression data in the TIMER database, we explored the correlation between MAP4 and EMT marker expression. Notably, in 515 lung adenocarcinomas, MAP4 protein levels were positively correlated with EMT-related protein levels (Fig. 8A). Thus, these data provide preliminary further indications of a positive correlation between MAP4 and EMT processes in TCGA LADC specimens. To further explore the association of MAP4 and EMT markers in LADC tissues at the organizational level, we used RT‑qPCR to detect the expression levels of *E*‑cadherin, *N*‑cadherin, Vimentin, Slug and MAP4 mRNAs in 54 LADC clinical samples. Consistent with bioinformatics results, MAP4 is positively correlated with the expression level of EMT markers (Fig. 8B). Next, we explored the viability of A549 and H1299 cells treated with MAP4 siRNA and IR (0, 2, 4, 6, and 8 Gy), and the results showed that the growth of MAP4-downregulated cell lines was significantly inhibited (Fig. 8C, D). To investigate the effect of MAP4 on the radiosensitivity of lung adenocarcinoma cells, 6 Gy X-ray irradiation was subsequently applied to determine the effect of MAP4 interference on EMT-related proteins. The results suggested that after 6 Gy X-ray irradiation, the expression of E-cadherin decreased and the expression of Vimentin increased in A549 and H1299 cell lines, but these effects could be reversed by MAP4 interference (Fig. 8E, F). The above results suggest that MAP4 can enhance the radiation resistance of lung adenocarcinoma cells. The effects of ionizing radiation on these EMT-related markers could be reversed by MAP4 gene interference. MAP4 is involved in the EMT process induced by ionizing radiation, and MAP4 is a potential target molecule that promotes EMT induced by ionizing radiation in lung adenocarcinoma cells.

## Discussion

Radiation therapy is a common and effective treatment for LADC. Despite advances in radiotherapy techniques, successful treatment of lung adenocarcinoma remains difficult due to the presence of radioresistance. Previous studies have shown that MAP4 can promote malignant cell proliferation, invasion and migration in various solid tumours. There is a significant association between high MAP4 expression and the presence of residual tumours in ovarian cancer. Low MAP4 expression was significantly associated with the lower stage. MAP4, Syk, and calpain-1 are interrelated and interact to regulate ovarian cancer cell proliferation (Ou et al. 2014). The level of MAP4 in HCC tissues was higher than that in normal liver tissues. MAP4 knockdown inhibited the migration and invasion abilities and EMT process of HCC cells. Further evidence suggested that the upregulation of β-catenin activity induced by the interaction between MAP4 and GSK3β might be responsible for the pro-migratory and pro-EMT effects of MAP4 on HCC cells. MAP4 promoted the migration, invasion and EMT of HCC cells by regulating the GSK3β/β-catenin pathway (Lamouille et al. 2014). MAP4 interacts with BRINP3 at the protein level, and overexpression of MAP4 can partially reverse the inhibitory effect of downregulated BRINP3 on osteosarcoma cell proliferation and invasion, suggesting that downregulation of BRINP3 may inhibit osteosarcoma cell proliferation and invasion by inhibiting MAP4 expression. The results indicated that BRINP3 functions as an oncogene within osteosarcoma through MAP4 and thus could be used as a potential biomarker for the diagnosis and treatment of osteosarcoma (Parker et al. 2014). Another study showed that dual silencing of IKBKB/STK39 or EDN2/TBK1 stabilizes microtubules by inhibiting the phosphorylation of p38 and MAP4, inducing apoptosis and blocking the cell cycle more effectively than silencing individual kinases. Knockdown of IKBKB/STK39 or EDN2/TBK1 enhanced paclitaxel sensitivity in two ovarian xenograft models (Kang et al. 2013). In addition, Thapa et al. used breast cancer cells as a model and found that MAP4 promoted cancer cell invasion through the PI3K/AKT signalling pathway (Permana et al. 2005). Since MAP4 has a strong influence on the dynamics of the cytoskeleton, especially on microtubules and microfilaments (Pollom et al. 2016), studies have reported that Tctex-1, regulated by MAP4, enhances epidermal cell migration and wound healing during hypoxia (Ramkumar et al. 2018). p38/MAPK signalling-induced MAP4 phosphorylation promotes angiogenesis by inducing proliferation and migration of endothelial cells cultured under hypoxic conditions through regulation of microtubule dynamics. Hyperphosphorylation of MAP4 (S737 and S760) through the p38/MAPK pathway accelerates the migration and proliferation of keratinocytes and the healing of skin wounds (Shintani et al. 2011; Singh et al. 2018). MAP4 expression is ubiquitous in nonneural samples and plays a key role in microtubule assembly in human cells. Researchers found that the ratio of MAP4 to Stathmin mRNA in NSCLC tissues was higher than that in normal specimens, suggesting that this ratio may be a potential prognostic marker in NSCLC patients (Singharajkomron et al. 2022). Luo et al. studied the relationship between the mRNA transcript level and protein expression level of different MAPs and related prognosis in NSCLC patients (Stanton et al. 2011). Another study identified transcriptomic biomarkers in MAP genes for lung cancer diagnosis and prognosis by analysing differential gene expression and its correlation with tumour progression (Su et al. 2015).

EMT is a process in which epithelial tumour cells lose epithelial polarity, adhesion, and motility and convert to a mesenchymal phenotype. EMT is characterized by the loss of epithelial morphology and the acquisition of mesenchymal morphology; a critical stage of the EMT process is the downregulation of epithelial markers (*E*-cadherin, desmoplakin, occludins, claudins and ZO-1) and upregulation of mesenchymal markers (*N*-cadherin, vimentin, and FSP1) by transcription factors and transcriptional repressors (Su et al. 2016; Sung et al. 2021). In fact, EMT was originally discovered and characterized during embryonic development, where it is essential for the differentiation of embryonic mesenchymal stem cells into a variety of cell types and tissues (Li et al. 2017). At the gene level, a variety of signalling pathways are involved in the EMT of tumour cells, and inhibiting the occurrence of EMT can inhibit cancer metastasis (Thai et al. 2021). An important factor in recurrence and metastasis is EMT, an abiotic process in which epithelial cells lose their polarity. During this process, cells are reprogrammed to a mesenchymal phenotype, losing their differentiated phenotype and tight cell‒cell junctions. Importantly, NSCLC cells undergoing EMT contribute to the development of malignancy. Furthermore, loss of E-cadherin may contribute to tumour radioresistance by affecting DNA repair and cell cycle checkpoints (Thapa et al. 2020). EMT also plays an important role in tumour aggressive behaviour, cancer stem cell properties, and resistance to chemotherapy, immunotherapy, and radiotherapy in various cancers (Vinod and Hau 2020; Wang et al. 2020; Xia et al. 2018). Radioresistance is affected by many factors, and EMT can induce radiation resistance in tumour cells (Xue et al. 2020; Yang et al. 2018). Accumulating evidence indicates that tumour metastasis mediated by EMT is closely related to radioresistance (Li et al. 2020; Yang and Weinberg 2008). Previous studies have reported that surviving NSCLC cells treated with ionizing radiation exhibit an EMT phenotype. EMT-induced NSCLC cells exhibit resistance to radiation (Yu et al. 2015; Yuan et al. 2020). It was also found that mesenchymal lung cancer cells exhibited higher levels of radioresistance than cancer cells of the epithelial phenotype (Yang and Weinberg 2008). More importantly, it was also observed that radiotherapy might induce EMT in vivo, as demonstrated by comparing surgically resected NSCLC specimens before and after radiotherapy (Zeng et al. 2022). More evidence suggests that IR-induced EMT accelerates the malignant features of radiotherapy-treated tumours, leading to recurrence and treatment failure of nasopharyngeal, breast, oesophageal, hypopharyngeal, and lung cancers by promoting invasion, migration, radiation resistance, and chemotherapy resistance (Li et al. 2020; Zeng et al. 2022; Zhang et al. 2016; Zhang et al. 2019a; Zhang et al. 2019b; Zhang et al. 2019c).

The results of our immunohistochemistry and RT‒qPCR showed that the protein and mRNA levels of MAP4 in LADC tissues were significantly increased compared with those in normal lung tissues, suggesting that MAP4 was highly expressed in LADC tissues. Both our clinical data and bioinformatic analysis of lung adenocarcinoma showed that increased MAP4 expression in LADC was significantly associated with poorer PFS and OS. Univariate Cox regression analysis showed that pT stage, pN stage, pTNM stage and MAP4 expression were all associated with poor OS in patients with lung adenocarcinoma. Multivariate Cox regression analysis revealed that only pT stage and MAP4 were independent prognostic markers for poor OS in LADC patients, suggesting that MAP4 may play an important role in the progression of LADC. In view of the prognostic value of MAP4 in LADC, according to the results of LASSO regression analysis, we established a nomogram prognostic model based on clinical factors and MAP4 expression. Through discrimination and calibration analyses, our nomogram was shown to be a robust prognostic model. The results of ROC and C-index indicated that the nomogram constructed by combining clinical factors and MAP4 in the training set had a higher AUC value and predictive ability than the nomogram of clinical factors alone. However, this tendency was worse in the validation set than in the training set, possibly related to the smaller sample size in the validation set. In addition, the DCA results also show that the model has a higher net benefit than existing models. After expanding the sample size, consistent with our previous findings, we found that MAP4 may act as an oncogene to affect the prognosis of lung adenocarcinoma. MAP4 function is associated with tumour metastasis or a poor prognosis in human cancers. The results of wound healing experiments and transwell experiments in this study showed that the downregulation of MAP4 significantly inhibited the migration and invasion of LADC cells, which confirmed that MAP4 can promote the migration and invasion of lung adenocarcinoma cells once again. MAP4 may worsen the prognosis of lung adenocarcinoma by promoting the migration and invasion of lung adenocarcinoma cells. These results also confirmed the results of previous reports on other cancer types (Kurrey et al. 2009; Kwon et al. 2017; Lamouille et al. 2014; Parker et al. 2014). Determining the underlying molecular mechanisms associated with radiation-induced EMT will help predict solid cancer recurrence and will greatly improve the treatment of this disease (Zhang et al. 2015; Zhou et al. 2020). Recent studies have shown that EMT plays a crucial role in the development of cancer radioresistance. However, to the best of our knowledge, no study has reported that MAP4 plays a radiosensitizing role in lung adenocarcinoma cells by affecting EMT. Since EMT is a critical step in cancer invasion and metastasis, we hypothesized that radiation-induced EMT and acquired radioresistance might be associated with MAP4. As determined by the CCK8 assay, the results suggested that the silencing of MAP4 is associated with the viability of lung adenocarcinoma cells. MAP4 protein levels were positively correlated with EMT-related protein levels in LADC samples from the TCGA database and our clinical tissue samples of lung adenocarcinoma. These results preliminarily suggest that there is a strong correlation between MAP4 and EMT. Our results showed that there was a correlation between the expression of MAP4 and the radioresistance of LADC cells, so we initially explored the possible mechanism of the relationship between MAP4 and radioresistance. Western blot analysis showed that the expression of E-cadherin increased in lung adenocarcinoma cells after radiation, while the expression of Vimentin decreased significantly, suggesting that the process of EMT was induced by radiation. However, in MAP4-silenced cell lines, the radiation-induced EMT process was partially reversed. In contrast, during LADC radiotherapy, MAP4 may enhance the EMT process through synergistic radiation, thereby increasing the resistance of LADC cells to radiation. Therefore, we speculate that MAP4 may be related to the acquisition of radioresistance in lung adenocarcinoma cells during radiotherapy through EMT.

This study has some limitations that need to be considered. First, retrospective studies may introduce unavoidable selection bias. Second, the nomogram was only validated by internal validation, and a multicentre external validation with a larger sample size is also needed. Therefore, a multicentre study is needed to verify the generalization ability of the proposed nomogram. Finally, the current study only preliminarily confirmed that MAP4 is involved in the radiation resistance of lung adenocarcinoma through EMT, and more research is needed to understand the underlying mechanism.

## Conclusion

In summary, our results suggest that MAP4 is overexpressed in lung adenocarcinoma; it worsens the prognosis of LADC by promoting the migration and invasion of lung adenocarcinoma cells. We developed a nomogram including clinical factors and MAP4 expression that can be used for prognosis prediction in patients with lung adenocarcinoma. MAP4 plays a crucial role in the development of radioresistance in lung adenocarcinoma by regulating the radiation-induced EMT pathway. MAP4 may serve as a biomarker for the prognosis assessment of lung adenocarcinoma and as a new therapeutic target for improving radiosensitivity.

## Data Availability

The datasets generated during and/or analysed during the current study are available from the corresponding author upon reasonable request.
